# ZT > 0.1 Electron‐Carrying Polymer Thermoelectric Composites with In Situ SnCl_2_ Microstructure Growth

**DOI:** 10.1002/advs.201500015

**Published:** 2015-05-08

**Authors:** Robert M. Ireland, Yu Liu, Xin Guo, Yu‐Ting Cheng, Srinivas Kola, Wei Wang, Toinetta Jones, Ronggui Yang, Michael L. Falk, Howard E. Katz

**Affiliations:** ^1^ Department of Materials Science and Engineering and Department of ChemistryJohns Hopkins University3400 North Charles StreetBaltimoreMD 21218‐2068USA; ^2^ Department of Mechanical EngineeringUniversity of ColoradoBoulderCO80309‐0427USA; ^3^ Western High School200 East North AvenueBaltimoreMD21202USA

**Keywords:** conducting polymer, Seebeck coefficient, thermal conductivity, thermoelectric, tin chloride

## Abstract

**An n‐type pyromellitic diimide polymer composite with in situ microstructure** growth of the common element compound SnCl_2_ reaches power factor of 50–100 μW m^−1^ K^−2^, the highest purely n‐type polymer composite power factor yet reported. The composite has a gigantic Seebeck coefficient between −4000 and −5000 μV K^−1^, many times higher than other polymer composites.

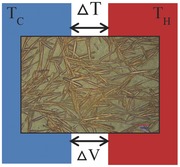

This is an open access article under the terms of the Creative Commons Attribution License, which permits use, distribution and reproduction in any medium, provided the original work is properly cited.

Solution‐deposited polymer‐based composites are interesting for ambient temperature cooling and subwatt power generation where sufficient power is more critical than high efficiency. Such composites combine solution processing, mechanical flexibility, and low thermal conductivity with sufficient power factor (PF), contributing to high figures of merit, ZT = *S* ^2^ *σT*/*κ*, where *S* is Seebeck coefficient, *σ* is electronic conductivity, *S* ^2^ *σ* is PF, *T* is absolute temperature, and *κ* is thermal conductivity.^1^, ^2^ Thermoelectric performance of hole‐carrying (p‐type) polymers has been enhanced so ZT exceeds 0.1.^3^^–^ 9 ZT of 0.1 would be an important milestone for a new class of materials, or for medical or mobile applications.

Krebs and co‐workers demonstrated that printing is the most rational choice for scalable manufacturing of large‐area energy saving devices.^10^ Yazawa and Shakouri and a few others compared polymer materials with conventional semiconductor materials in terms of power per unit mass and unit cost.^11^, ^12^, ^13^ Polymers were found valuable for lightweight remote power generation for sensors in conjunction with passive heat sinks (utilizing low heat flux).

Most prior work in polymer thermoelectrics was on p‐type polymers, especially poly(ethylenedioxythiophene) (PEDOT), sometimes mixed with heavy element compound semiconductors, having PF values >100 μW m^−1^ K^−2^ that lead to ZT > 0.1, the highest reported for polymers,^3^, ^4^, ^5^, ^6^, ^7^, ^8^, ^9^ perhaps as great as 0.4 when doping is optimized.^14^ There are far fewer reports of electron‐carrying (n‐type) thermoelectric polymers, also critical for thermoelectric modules. Fullerenes and powder‐processed organometallic poly(Ni 1,1,2,2‐ethenetetrathiolate) derivatives have shown high thermoelectric performance.^15^, ^16^, ^17^, ^18^, ^19^ Zhu and co‐workers^19^ achieved PFs from 6 to 60 μW cm^−1^ K^−2^ (*S* around −100 to −150 μV K^−1^, and conductivities of 5–40 S cm^−1^) from insoluble metal coordination n‐ and p‐type structures, leading to ZT near 0.1 at ambient temperature and 0.2 at 130 °C. Results on solution‐processable materials have been on imide‐containing polymers, or imide‐based small molecules, without inorganic additives.^20^, ^21^, ^22^ The first was an n‐type polymer prepared by our group; *S* was around −40 μV K^−1^.^20^ Schlitz et al. demonstrated solution doping of a high mobility n‐type polymer, poly[*N*,*N*′‐bis(2‐octyl‐dodecyl)‐1,4,5,8‐napthalenedicarboximide2,6‐diyl]‐alt‐5,5′‐(2,2′‐bithiophene)] (P(NDIOD‐T2), using dihydro‐1H‐benzoimidazol‐2‐yl (N‐DBI) derivatives as potential dopants,^21^ achieving electrical conductivities of nearly 0.01 S cm^−1^ and PF of 0.6 W m^−1^ K^−2^. Segalman and co‐workers showed record‐high thermoelectric performance for solution‐processed perylene diimide molecules, resulting in *σ* of 0.4 S cm^−1^, *S* around −180 μV K^−1^, and a PF of 1.4 μW m^−1^ K^−2^.^22^

We and others have sought soluble additives for n‐type polymers that could provide n‐type thermoelectric activity and doping capability, the latter a challenge in itself.^21^, ^22^, ^23^, ^24^ We expect composites to have improved thermoelectric performance due to enhanced electron conduction pathways, enhanced polymer doping, additional electronic states at the domain interfaces, along with low thermal conductivity. Polymer‐assisted microstructure growth has been shown to strengthen interfacial interactions, including electronically driven interactions between polymer and as‐grown particles. For example, cadmium telluride nanocrystals were synthesized in poly(3‐hexylthiophene) without surfactants for photovoltaic applications.^25^ Spectroscopy shows that the particles are bound to the polymer via dipole–dipole interactions and form a charge transfer complex. We chose SnCl_2_ as an additive because of its well‐established reducing activity in organic chemistry. Surprisingly, its electronic properties as an anhydrous solid do not appear to have been measured, nor has its in situ microcrystallization in poly­mer environments been observed; the closest precedent being hydrated proton conductors.^26^, ^27^ Other tin‐based materials have previously been investigated as potential TE materials, experimentally and through computation, including pure tin films, tin oxides, tin selenides, tin clathrates, and tin sulfide, or as beneficial TE impurities in telluride‐based inorganic materials.^28^, ^29^, ^30^, ^31^, ^32^, ^33^, ^34^, ^35^, ^36^, ^37^, ^38^, ^39^

We now show that a form of our original pyromellitic diimide (PyDI) polymer, with pentafluorophenyl end caps (thus abbreviated PyDI‐5FPE), mixed with in situ–crystallized SnCl_2_, forms a particularly effective platform for n‐type polymeric thermoelectric materials. We compare this system with a commercial n‐type polymer, P(NDI2OD‐T2) (Polyera N2200), and with analogous p‐type polymer systems (PQT12 and PBTTT with cobalt(III) acetylacetonate (Co(acac)_3_)) to show more broadly that weakly doping microstructured inclusions can act synergistically with both n‐ or p‐type polymers to give enhanced thermoelectric performance. **Figure** **1**a shows the chemical structure for the polymers.

**Figure 1:**
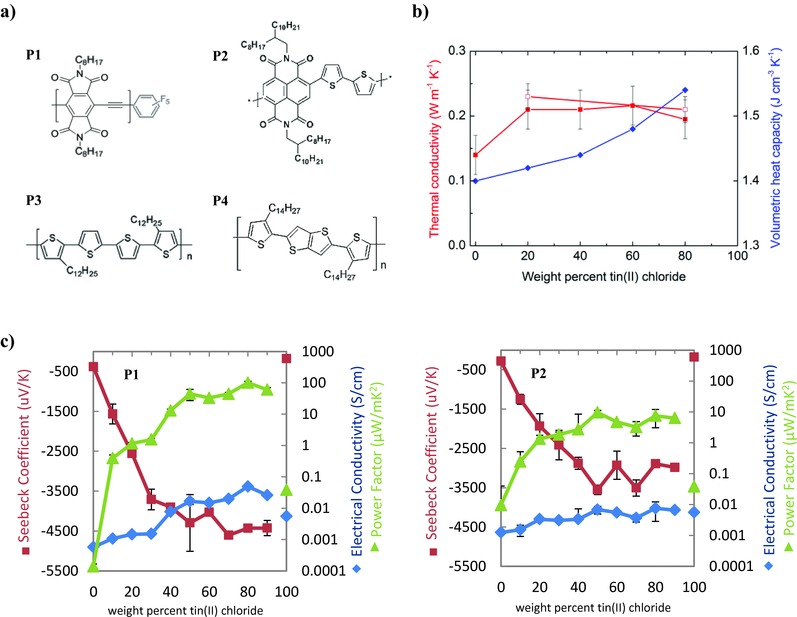
a) Chemical structure of poly(PyDI‐ethynylene)‐5FPE (**P1**), P(NDI2OD‐T2) (**P2**), PQT12 (**P3**), and PBTTT‐C14 (**P4**). b) Thermal conductivity and heat capacity versus weight% SnCl_2_ in **P1** (solid points) and **P2** (open squares) films. c) The Seebeck coefficient, electrical conductivity, and power factor are plotted versus concentration of initial tin(II) chloride precursor within **P1** (left) and **P2** (right) polymer matrices. Values are the average of at least ten samples. Error bars are standard deviations.

We added SnCl_2_ from 0 to 90 wt% to **P1** and **P2**, separately. Incorporation of SnCl_2_ and subsequent drop‐casting or spin‐casting on glass resulted in spontaneous formation of tin‐based microcrystals within the **P1** polymer matrix during solvent evaporation. **Figure** **2** shows laser optical images of SnCl_2_ in PyDI‐5FPE for concentrations 20–90 wt%, and for pure SnCl_2_ films. The pure SnCl_2_ films were processed exactly the same way as composites, meaning that the powder was dispersed in organic solvent but not mixed with polymer, just drop‐cast or spin‐coated onto the substrate utilizing the same conditions.

**Figure 2:**
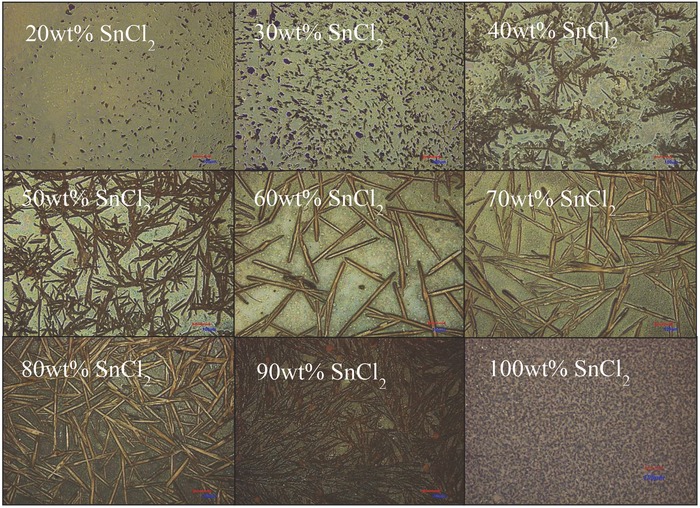
Optical images of SnCl_2_ microstructure captured in **P1** polymer matrix by drop‐casting from starting concentrations ranging 20–90 wt%, and of pure SnCl_2_ drop‐cast from solution on glass alone. Scale bars are all 100 μm.

**P1** is particularly effective at promoting, or allowing, SnCl_2_ micro/nanostructure growth. Elongated microcrystals can be observed for starting SnCl_2_ concentrations above 30 wt%, having aspect ratios >10. By contrast, only well‐dispersed globular clusters are obtained in **P2**. The particles obtained from initial concentrations of 60–80 wt% SnCl_2_ blended with **P1** resulted in the most prominent shapes (sharper interfaces) and greater monodispersity, reaching widths of 10–40 μm and lengths of 100–400 μm. Spin‐casting resulted in very well‐dispersed particulates, having significantly reduced dimensions, but increased homogeneity and still showing rod‐ and barlike structures. Optical images for 40, 60, and 80 wt% SnCl_2_ samples obtained by spin‐casting are shown in Figure S1 (Supporting Information).

Clusters and elongated crystals grown in the polymer matrix both appear to be mostly SnCl_2_, possibly with some metallic tin and tin oxide, according to energy dispersive spectroscopy (EDS) utilizing a scanning electron microscope (SEM), X‐ray photoemission spectroscopy (XPS), and X‐ray diffraction (XRD). EDS shows that crystals have high SnCl_2_ content but also have some oxide presence, and that there is essentially no difference in the composition of different structures/shapes (clusters vs crystals vs pure film). Elemental maps of particles in polymers obtained by EDS can be found in Figures S2 and S3 (Supporting Information). We observe that the SnCl_2_ additive is dispersed homogeneously within the polymer at a molecular level in addition to the secondary phase particulates at micrometer scale that are more easily observed. XPS suggests SnCl_2_‐in‐polymer has decreased work function, consistent with preliminary scanning Kelvin probe microscopy (KPM) and ultraviolet photoelectron spectroscopy (UPS) results shown in Figures S4 and S5 (Supporting Information). KPM shows that the poly­mer chemical potential is pulled toward vacuum and SnCl_2_ is pushed away from vacuum at the interface, which suggests that electrons accumulate in the polymer at their interface, with negative end of the interfacial dipole within the polymer. UPS shows that charge transfer occurs by evidence of an extra broad peak at lowest binding energy (Figure S5, Supporting Information). This is a direct observation of the interfacial charge transfer and new states that lie within the polymer bandgap, where electrons generated at the polymer–inorganic interface can reach the surface and contribute to the UPS signal.

Small XRD peaks can be indexed to tin metal and tin oxide, demonstrating that the obtained product is composed of multiple hybrid structures and interfaces (Figure S6, Supporting Information). The polymer‐grown tin structures formed in **P1** exhibit very similar phases (Figure S7, Supporting Information).

Thermal conductivity *κ* of polymers and composites was measured using the femtosecond laser‐based frequency‐dependent transient‐thermoreflectance method in conjunction with a multiple location and multiple spot‐size sampling technique (see the Supporting Information).^40^ The *κ* we measured is 0.14 W m^−1^ K^−1^ for both **P1** and **P2** with an uncertainty of 0.02 W m^−1^ K^−1^ while the heat capacity is 1.40 J cm^−3^ K^−1^ with an uncertainty of 0.2 J cm^−3^ K^−1^. The measurements on drop‐cast SnCl_2_ composites indicate that values of *κ* are only slightly increased relative to pristine polymers for both **P1** and **P2**, converging on 0.2 W m^−1^ K^−1^ for all compositions as the area sampled is increased to cover representative contributions from both polymer and SnCl_2_ components (Figure 1b and Figure S10, Supporting Information). The thermal conductivity of composites depends on the volume fraction, morphology, the thermal conductivity of the polymer and the inorganic crystals, and the interfacial thermal conductance.^41^, ^42^, ^43^ Although the thermal conductivity of SnCl_2_ is not available in the literature, we expect the value to be on the order of 0.3–0.6 W m^−1^ K^−1^, which is consistent with many similar crystalline dichlorides.^44^, ^45^ In addition, we observed maxima of 0.5 W m^−1^ K^−1^ in Figure S10 (Supporting Information), which would be close to the thermal conductivity of SnCl_2_ itself. Considering that the microstructure is relatively large, on the order of a few micrometers, based on the models cited,^41^, ^42^, ^43^ the contribution of the intrinsic interface resistance might be negligible in these composites. The comparison of the measured data with the Nielson model is presented at the conclusion of the Supporting Information. While this model appears to fit reasonably for the lower SnCl_2_ volume fractions, it is likely not suitable for the highly anisotropic crystallites formed at higher SnCl_2_ concentrations.

The Seebeck coefficient *S* was evaluated using standard thin‐film thermoelectric measurement techniques, taken on a homemade setup, utilizing preferred electrode geometry that reduces measurement error.^46^ The sample is mounted between a pair of Peltier heater–cooler tiles, with one electrode of the sample over each tile. Thermal EMF (Δ*V*) and the temperature difference (Δ*T*) were measured simultaneously by probing the pair of electrodes with a source meter and thermocouples. We used an optimal thin‐film electrode geometry (being a set of narrow, parallel strips, and no semiconductor material outside of the interelectrode region or space between electrodes), which minimizes external conduction pathways. In order to minimize signal‐to‐noise and overcome the increased device impedance associated with this otherwise preferred electrode arrangement, gallium indium eutectic was used on the voltage probe contacts to minimize contact resistance. Thermal paste is used to minimize thermal contact resistance between the bottom of our substrates and Peltier devices (Figure S8, Supporting Information). Total electrode length is >10 mm and distance between electrodes is ≈2 mm, where the effective width of the electrodes is the area covered by semiconductor, which is also ≈2 mm. The resulting ratio of aspect ratio for the channel to the aspect ratio of the electrodes is close to or less than unity, ensuring small relative errors and minimal overestimation of *S*.^46^ More precise measurements become difficult because the aspect ratio of the electrodes should be greater than the channel aspect ratio for accurate *S* measurement, although thin channel widths are required for low signal‐to‐noise and for accurately measuring resistance of low‐conductivity materials due to device impedance. Sheet resistance was measured by four‐probe technique with gallium indium eutectic applied to the probe tips to further minimize contact resistance and to mimic *S* measurements, and the electrical conductivity was calculated using poly­mer film thickness of each device. All devices are tested under ambient conditions in air, but protected from light and thermal convection.

Figure 1c shows *S*, *σ*, and PF as function of SnCl_2_ concentration in polymer matrices **P1** and **P2** prepared by drop‐casting. The values are averages over at least ten samples, taken from 3 to 5 repeated experiments, balancing statistical significance with the consideration that it takes about 1 h for each run. **P1** shows modest *σ* in its undoped form (0.00057 S cm^−1^), and a significant *S* (−380 μV K^−1^). **P2** shows greater *σ* (0.00124 S cm^−1^), as expected due to higher intermolecular overlaps of diimide cores, resulting in much greater PF despite its lower intrinsic *S* (−280 μV K^−1^). PFs are 0.00014 and 0.0095 μW m^−1^ K^−2^ for **P1** and **P2,** respectively. Our PF value for the pristine **P2** is almost one‐tenth the value recently reported in a doped **P2** system.^21^

SnCl_2_ evaporated onto glass, nominally 50 nm thick, shows PF an order of magnitude greater than does drop‐cast SnCl_2_, about 0.16 and 0.038 μW m^−1^ K^−2^, respectively. Evaporated and drop‐cast pure SnCl_2_ both show *σ* around 4–5 × 10^−3^ S cm^−1^, but the *S* is apparently increased by a factor of five when sublimed (which presumably forms more pure and densely connected crystalline solids), reaching about −530 μV K^−1^. The sublimed and drop‐casted materials both form films comprising multiple structures (Figure S9, Supporting Information).

PFs of our hybrid composites are greater than those of any of the individual components, reaching 50–100 μW m^−1^ K^−2^ for SnCl_2_ precursor concentrations above 50 wt% blended with **P1**. Thus, estimating PF of 80 μW m^−1^ K^−2^ for 50–80 wt% SnCl_2_, *T* of 300 K, and *κ* of 0.2 W m^−1^ K^−1^, we project ZT of 0.12. PF and *κ* are constant within experimental error over this range, and thus ZT is as well. PF, and thus ZT, is about one‐tenth as large for **P2** composites. It appears that in situ microstructure growth of SnCl_2_ enormously increases *S*, and possibly *σ* of the polymer. *S* increases significantly for **P1** with increasing concentration of SnCl_2_, saturating around −4500 μV K^−1^ above 50 wt% SnCl_2_. *σ* increases only slightly for **P1** hybrids with low concentrations of SnCl_2_. *σ* increases sharply between 30 and 50 wt% and remains constant up to 90 wt% SnCl_2_, leveling at about 0.05 S cm^−1^. A strong increase in electrical conductivity appears to be correlated with overlapping of the elongated Sn‐containing crystals, as they reach sufficient size to create a percolated network above 40 wt% SnCl_2_. Utilizing **P2** as the polymer host matrix showed a similar trend as **P1** blended with SnCl_2_, but lower PFs closer to 10 μW m^−1^ K^−2^ were obtained. Though with a much lower *σ*, the PFs are on par with high‐performance p‐type hybrid composites.

Contributions of static electricity on an electrically isolated probe or highly resistive contacts to the very large Seebeck coefficients we measured are ruled out by the electrode and probing arrangements. As long as probes are small enough to be at constant temperature over their whole area, then the largest temperature difference on the sample must be across or within the interelectrode region, and we meet these criteria. The fact that we obtain obvious trends of *S* as a function of material composition is further evidence that the data reflect material rather than contact properties.

Our results on n‐polymers with inorganic additives prompt similar experiments using hole‐carrying polymers PQT12 (**P3**) and PBTTT‐C14 (**P4**). The weak dopant Co(acac)_3_ will form large crystals readily in p‐type polymers such as **P3** and **P4**. *S* > 2000 μV K^−1^ and power factors of 10 μW m^−1^ K^−2^ were obtained, with slight increase in hole conductivity (from 0.0013 to 0.021 S cm^−1^). Different morphologies, including elongated uniaxial microcrystals, were demonstrated using 20, 40, and 60 wt% additives within **P3** polymer matrix, as seen in **Figure** **3**. Similar features were obtained for Co((acac)_3_) blended with **P4**, which are included in Figure S11 (Supporting Information). The results of *S*, *σ*, and PF plotted for **P3** blended with Co((acac)_3_) as a function of dopant concentration are also shown in Figure S12 (Supporting Information). We anticipate considerably higher power factors as doping capacity of the inorganic phase is increased, by either using stronger dopants or obtaining more homogeneous dispersion by arresting particle growth at nanoscale dimensions. Further detailed exploration of metal acac‐*p*‐polymer systems will be published separately.

**Figure 3:**
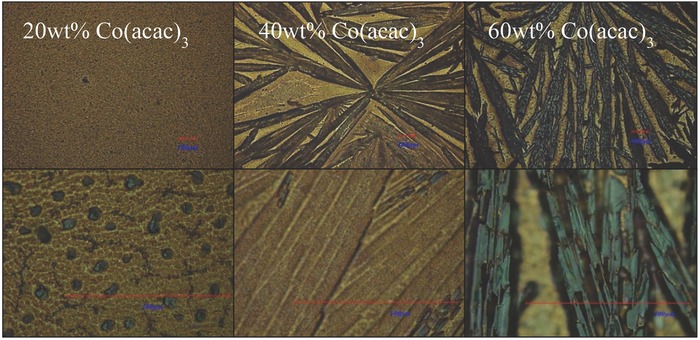
Optical microscope images of **P3** (PQT12) blended with 20, 40, and 60 wt% cobalt(III) acetylacetonate (top and bottom from left to right). Scale bars are all 100 μm.

In situ fabrication of inorganic particles within the polymer matrix is advantageous because of simpler preparation and improved interfacial interactions,^25^ and better morphology.^4^

There are several competing arguments for either macrocrystals or nanosized inclusions being more desirable. First, nanostructuring increases surface area to volume ratio greatly at a cost of increasing contact resistances.^9^ Although microcomposites are generally less homogeneous compared to nanocomposites, they may have greater conductivity because larger inclusions may have greater carrier mobilities due to longer grain size and thus potentially longer mean‐free paths. Smaller nanoscale dopants can also have high tendency to diffuse in organic systems, leading to detrimental instability.^47^ However, phononic thermal conductivity is greatly reduced due to the abundance of interfaces achieved by nanostructuring. The control of particle growth (i.e., growth rates, crystal structure and orientation, and particle alignment) leading to optimized power factors and reduced phononic thermal conductivity would be essential for further increases in composite ZT.

The platform described above offers numerous opportunities to increase ZT further, especially for n‐type polymer composites. *S* and *σ* can increase simultaneously in our composites because of the inorganic compound introducing some new filled states to the polymer besides having some conductivity itself. Simple additions of n‐doping elements such as sulfur and aluminum to SnCl_2_ should increase *σ*. This should not result in accompanying increase in *κ* (Figure S18, Supporting Information) because we would remain in the phonon thermal conductivity–dominated regime. Growth of precise nanostructures will increase *S* and *σ* while decreasing *κ* further if it allows maximum interface phonon scattering while maximizing doping and transport pathways.

Our use of a novel unipolar n‐type polymer and inorganic additives has led to the first demonstration of ZT > 0.1 in electron‐carrying polymer composites that contain neither highly toxic nor rare elements. Leveraging recent advances in n‐poly­mer doping, we anticipate equivalent power factors from both composite polarities to be achievable in the near future.

## Experimental Section

Microscope slides (Corning Inc.) were cleaned by sonication in deionized water, acetone, and 2‐propanol for 10 min each, and blowing dry with N_2_. Electrode contacts were predeposited by thermally evaporating Au or Al from alumina crucibles using an Edwards thermal evaporation system (50 nm thick, pressure below 5 × 10^−6^ Torr, deposition rate 0.5 Å s^−1^). Deposition rates and thicknesses were monitored by quartz crystal microbalance.

Polymer and inorganics were weighed in separate glass vials and made up to 10 mg mL^−1^. The polymer was placed on a hot plate (80 °C) while solutions of inorganic were sonicated (30 min). The polymer was blended in the desired concentration with inorganic solutions using a pipette, and vigorously stirred (10 min) before drop‐casting. The final blend was dropped by pipette into 2D wells, which were fabricated by laying a pattern of Novec fluorinated polymer, on the glass substrates having predeposited metal electrodes. The result was a square cm film with 1–2 μm thickness, lying over two metal electrodes with a channel gap (each about 0.2 cm across and 1 cm long). The solvent was allowed to evaporate overnight while samples were kept in a petri dish in the fume hood. Next, samples were placed in vacuum oven overnight (100 °C under low vacuum) to remove residual solvent. Just before taking electrical measurements, the films were heated under nitrogen atmosphere in the oven (150 °C for 10–30 min).

Compositional and structural analysis of all **P1**‐based composites was carried out by X‐ray photoemission spectroscopy, X‐ray diffraction, and by energy dispersive spectroscopy utilizing the backscatter detector of a JEOL scanning electron microscope. Scanning Kelvin probe microscopy, a form of atomic force microscopy, was utilized to observe energy‐band alignment and vacuum level offset between material domains.

Regarding Seebeck coefficient measurements, numerous measurements of Δ*V*, typically 500, were made for each value of Δ*T*, with a standard deviation of 1–5% (0.01–0.05 mV) per data set. These Δ*V* were averaged to eliminate the noise signal induced by the environment and obtain just the consistent steady‐state value. Six incremental Δ*T* were imposed on the sample, so the slopes of Δ*V* versus Δ*T* give values of the Seebeck coefficient. The linearity of the data of Δ*V* and Δ*T* was used as a key criterion to ensure valid measurements. The setup was checked using Ni metal ingot or Te thin films deposited from vapor, for which the values we obtained (−19.8 ± 0.8 μV K^−1^ for Ni, and ≈400 μV K^−1^ for Te) agreed well with those reported. Resistivity measurements were employed using four‐probe measurement method with an Agilent 4155C Semiconductor Parameter Analyzer using low‐resistance probes (Micromanipulators). Thermal conductivity was measured with a time‐domain thermoreflectance (TDTR) system using femtosecond lasers (details are described in the Supporting Information).

## Supporting information

As a service to our authors and readers, this journal provides supporting information supplied by the authors. Such materials are peer reviewed and may be re‐organized for online delivery, but are not copy‐edited or typeset. Technical support issues arising from supporting information (other than missing files) should be addressed to the authors.

Supplementary
