# Supplementary material for: ZT > 0.1 Electron‐Carrying Polymer Thermoelectric Composites with In Situ SnCl2 Microstructure Growth
Source: Adv Sci (Weinh). 2015 May 8;2(6):1500015. doi: 10.1002/advs.201500015 (PMC5115405; doi:10.1002/advs.201500015)
Supplement: Supplementary file 1 — Supplementary [file ADVS-2-0r-s001.pdf]

Copyright WILEY-VCH Verlag GmbH & Co. KGaA, 69469 Weinheim, Germany, 2015.

ADVANCED SCIENCE
Open Access

Supporting Information

for *Adv. Sci.*, DOI: 10.1002/advs. 201500015

ZT > 0.1 Electron-Carrying Polymer Thermoelectric Composites with In Situ $SnCl_2$ Microstructure Growth

*Robert M. Ireland, Yu Liu, Xin Guo, Yu-Ting Cheng, Srinivas Kola, Wei Wang, Toinetta Jones, Ronggui Yang, Michael L. Falk, and Howard E. Katz**

Copyright WILEY-VCH Verlag GmbH & Co. KGaA, 69469 Weinheim, Germany, 2013.

Supporting Information

ZT > 0.1 Electron-Carrying Polymer Thermoelectric Composites with in-situ $SnCl_2$ Microstructure Growth

Robert M. Ireland, Yu Liu, Xin Guo, Yu-Ting Cheng, Srinivas Kola, Wei Wang, Ronggui Yang, Michael L. Falk, Toinetta Jones, and Howard E. Katz[*]

Composition and Structural Analysis of Polymer-inorganic Composites

We employed X-ray photoemission spectroscopy (XPS) on 60, 80, and 100 wt% $SnCl_2$ samples utilizing the pyromellitic diimide polymer with pentafluorophenyl end cap (PyDI-5FPE) to determine the abundant bulk atoms in our films (Figure S3).[1-3] All binding energies are referenced to the C 1s peak at 284.7 eV. For polymer hybrids, significant peaks for C (C=O) and F are present, indicative of the polymer matrix. Also the polymers show significantly less bound chlorine, which is consistent with more oxide growth. The peak for O 1s is observed at 529-532 nm. For pure Sn(II)Cl films, the peak lies at 531 eV, which suggests most oxygen detected is from nonstochiometric oxide, or oxygen vacancies (consistent with Sn^{4+}). The hybrid samples are similar but with peaks shifted towards 529 eV, suggesting more stochiometric lattice-bound oxygen (typically consistent with Sn^{2+}). Chlorine was more easily detected in the surface of the pure tin chloride film. Due to the surface sensitivity (0-10 nm), we are unable to quantify $SnCl_2$ in the polymer-grown crystals themselves (which EDS indicates are largely $SnCl_2$, possibly "buried" under oxide or polymer).

The characteristic Sn $3d_{5/2}$ peak is observed at 484-488 eV, and shows similar peak shift behavior as O 1s spectra when comparing pure $SnCl_2$ films with hybrid polymer films (similar shift for Cl 2p and C 1s peaks). For pure Sn(II)Cl we observe the peak at 487 eV and for the hybrids we see a shift to lower binding energy between 485 and 486 eV. The 487 eV peak has been reported widely as the characteristic peak for SnO_2, while 486 eV is more consistent with SnO and 484 eV for metallic tin. Unfortunately, these results cannot be used to determine structure, but we do know the metallic constituents (SnCl2 and oxide) have lower work function when grown within the polymer (shift to lower binding energies) and that there is an interaction between the polymer and inorganic (increase in peak-to-peak distance). Furthermore, the single peaks of Sn 3d5/2 and Sn 3d3/2 could be divided into multiple peaks, which could be attributed to metallic Sn, or Sn bonded to chlorine, oxygen, or carbon.

The slight shift in XPS peaks to lower binding energy could correspond to multiple effects. The literature shows that lower binding energy correlates to a lower bulk work function (lower oxidation states). Although the compositions are almost exactly similar, the work function could be shifted due to difference in coordination and bonding characteristics of Sn with the polymer (or in different oxide configurations, SnO_2 has greater work function than SnO has greater work function than Sn). It may also be evidence that the Fermi level of the inorganic bends upward at the interface with other semiconductors (i.e. the polymer matrix). It is possible

that the Sn(II)Cl films are mostly tin and oxygen-deficient SnO_2, and the elongated particles grown *in situ* of the polymer are likely to be more crystallized versions of the same. Another issue is that the composites are not pure, but yield mixed structures. XPS is consistent with X-ray diffraction and the presence of multiple well-distributed phases for $SnCl_2$/PyDI-5FPE composites.[4-8]

The crystals grow out of or on top of the polymer, possibly breaching the surface of the final resting polymer. For drop-cast composites, where the final polymer thickness is 1-2 μm, the crystals can show heights of 5 μm. Profiles of $SnCl_2$/PyDI-5FPE composites obtained by drop- and spin-casting are shown in Figure S13. The surface roughness and profile were visualized and measured using a laser optical microscope. There also appears to be clear visual evidence for strain induced in the polymer at boundaries with grown particles. It remains uncertain where or how $SnCl_2$ diffuses within the polymer and at which interfaces/sites it preferentially nucleates and grows. For spin-cast samples we obtain much greater uniformity and less surface roughness, as the thickness is lower and particles are captured well within the more compacted matrix (i.e. less diffusion and crystal growth is possible due to increased evaporation rates of solvent evaporation and film densification). The results of S, σ, and PF plotted as a function of dopant concentration for $SnCl_2$/PyDI-5FPE composites obtained by spin-casting are shown in Figure S12.

The non-uniformity in composite film thickness (possibly thicker in the crystal and thinner in polymer) adds uncertainty to both electrical and thermal conductivity measurements. However, as conductances add reciprocally (as sums of resistances) and the best performing films are <40% covered by crystals; the conductances would be overestimated by <30%, and the σ/κ ratio would not be affected, assuming that the inorganics do not form a percolating network. However, even if they do, our measurement of σ on a film of 100% $SnCl_2$ is not markedly different from measurements on composites.

Frequency-Dependent Time-Domain Thermoreflectance (TDTR) Measurement

Figure S15 (a) shows the schematics of the transient thermoreflectance (TTR) system using femtosecond laser to measure the thermal conductivity of the composite films. Similar to the implementation of most transient time-domain thermoreflectance (TDTR) systems,[6-8] a Ti:Sapphire laser oscillator is used, which outputs a train of 800 nm light pulses with a duration of 150 fs and a repetition rate of 80 MHz. The output laser is split into a pump beam and a probe beam. The wavelength of the pump beam is converted from 800 nm to 400 nm through second-harmonic generation. The pump beam passes through an electro-optical modulator (EOM) that modulates the beam at a frequency between 0.1 and 20 MHz, which is also input as the reference frequency for the lock-in detection of a weak signal. A mechanical delay stage changes the optical path length of the probe beam, which controls the time interval between the arrivals of a pump pulse and a probe pulse onto the surface of the sample. The pump and the probe beams enter collinearly into a 10x objective lens. An f = 300 mm lens and a large-active-area Si detector (3.6 mm * 3.6 mm, DET36A, Thorlabs) are used to collect the reflected probe beam. The locations of the objective lens and the Si detector are adjusted to vary the spot sizes of the pump and probe beams arriving at the samples. An inductor and a pre-amplifier are used to amplify the thermoreflectance signal.

The sample is prepared by depositing an Al transducer layer (~ 100 nm) on top of the target film via magnetron sputter deposition (Figure S15 (b)). The pump laser pulse heats the Al film and the evolution of the surface temperature is measured by the time-delayed probe pulse

through the temperature dependence of the optical reflectance, i.e. the thermoreflectance. The thicknesses of all our target films far exceed the penetration depth (d) of the thermal waves inside these films: $d = \sqrt{\frac{\kappa}{2\pi f C}}$, which ensures that the supporting substrate can be excluded from the heat transfer model. At f = 1 MHz for SiO_2, d = 0.36 μm while the thickness of SiO_2 is 2.2 μm. For polymethylmethacrylate (PMMA) and PyDI-5FPE and PyDI-5FPE composites mixed with *in situ*-crystallized $SnCl_2$, the thermal penetration depth d is around 0.14 μm. The thickness of PMMA is 500 nm, and thicknesses of the PyDI-5FPE and $SnCl_2$ composites (having $SnCl_2$ concentration 0-90 wt%) are ~1 μm.

Figure S16 shows the experimental and the modeled data for drop-cast (a) pure PyDI-5FPE and (b) 20 wt% $SnCl_2$ in PyDI-5FPE at three different modulation frequencies using a pump spot of r = 9.0 μm and a probe spot of r = 7.4 μm. The "Ratio" stands for the quotient of the real and imaginary parts of the probe signal output by the lock-in amplifier. We use the heat transfer model[6,9] that is solved in cylindrical coordinates for layered structures to calculate the "Ratio" as a function of delay time. In the model, there are three input parameters (the thickness, the volumetric heat capacity and the thermal conductivity) for each of the three layers (Al, interface and the target film where the target film is considered as semi-infinite since the thickness is much larger than the thermal penetration depth). All parameters are fixed by either literature values or independent measurements, except that the thermal conductivity of the target film is adjusted to obtain the best-fit result to the experimental data. The thermal conductivity of pure PyDI-5FPE is 0.14 ± 0.02 W m^{-1} K^{-1} while that for 20 wt% $SnCl_2$ in PyDI-5FPE is 0.23 ± 0.02 W m^{-1} K^{-1}.

Thermal Conductivity Sampling Technique

As shown in Figure 2 (in the main text), the hybrid composite samples are heterogeneous with inorganic $SnCl_2$ domain size on the order of 10-100 μm. This domain size is comparable or larger than the commonly-used laser spot sizes for transient thermoreflectance experiments, a few to 20 μm. For heterogeneous samples, the measured thermal conductivity is very likely dependent on the sampling location and the sampling size. The measured thermal conductivity is more likely to represent that of the entire sample if the sampling size is large enough. Therefore we expand the laser spot sizes to determine the asymptotic value of the thermal conductivity. To ensure that the asymptotic value does not vary from one location to the other, we repeat the multiple-spot-size experiment at four different locations of the sample.

The different laser spot sizes are obtained by placing the sample at different distances from the focal plane of the objective lens, where the beam waists of both the pump and the probe are located. The different distances are 60, 100, 150, 200, 250, 300, 400, and 500 μm, and the sampling size (R_{pp}), defined as the root sum square of the pump rms radius and the probe rms radius, increases linearly from 11.7 to 70.9 μm. These spot sizes of the pump and the probe beams are calibrated using the knife-edge method, where the sharp edges of a piece of Al-coated Si wafer are used in place of the knife edge. For both the calibration and the measurement, the location of the beam waists is found by maximizing the thermoreflectance signal at a fixed delay time.

We validate the multiple-spot-size method by measuring the thermal conductivities of two standard samples, namely, SiO_2 and PMMA, and a pure PyDI-5FPE sample as a function of the sampling size. As shown in Figures S17, the thermal conductivities of these homogenous samples remain constant with varying spot sizes. The experimental details and the fitting

parameters used in the thermal model are given below. For a sampling size of R_{pp} = 11.7 μm, we measure the data at three modulation frequencies (1.73, 2.46 and 3.23 MHz) to verify that the measured thermal conductivity is accurate. At other sampling sizes, we measure the data only at 1.73 MHz. To find the thermal conductivity of the samples, we take the volumetric heat capacities of Al, SiO_2, PMMA and PyDI-5FPE at room temperature to be 2.42, 1.67, 1.60 and 1.40 J cm^{-3} K^{-1} as the inputs for the heat transfer model.[11,12] We use cross-sectional SEM to determine the thickness of the Al transducer on a piece of Si wafer that is prepared at the same time with the other samples, at a precision of 2 nm. The thermal conductivity of Al can be calculated from its electrical conductivity using the Wiedemann-Franz law, which is measured using the four-point probe technique.

Thermal Conductivity of $SnCl_2$/PyDI-5FPE composites

After the system and the experimental procedure are calibrated, we apply the similar multiple location and multiple spot-size sampling procedure to measure the thermal conductivity of the hybrid $SnCl_2$/PYDI-5FPE composites with different percentage of $SnCl_2$.

To avoid damage to the polymeric materials, we use small laser powers for the pump and the probe, which increase linearly with sampling sizes. When R_{pp} is equal to 11.7 μm, we use 2 mW for the pump and 1 mW for the probe. When R_{pp} is equal to 70.9 μm, we use 12 mW for the pump and 6 mW for the probe. As the composite and PyDI-5FPE thicknesses are both 1 μm, the steady-state temperature rise is 7 K at R_{pp} = 11.7 μm and 1 K at R_{pp} = 70.9 μm. The thickness of PMMA is 500 nm so the steady-state temperature rise in PMMA is half as small as that in PyDI-5FPE. The transient temperature rise of the Al film created by the absorption of a single pump pulse is negligible.

To fit the experimental data for the thermal conductivity, the simple mixing rule is used to determine the volumetric heat capacities of the polymer-inorganic composites as the inputs for the heat transfer model. The densities of PyDI-5FPE and $SnCl_2$ crystal are 1 g cm^{-3} and 3.90 g cm^{-3}.[11] The volumetric heat capacities of PyDI-5FPE and $SnCl_2$ crystal are 1.40 J cm^{-3} K^{-1} and 1.68 J cm^{-3} K^{-1}.[10] Therefore for 0, 20%, 40%, 60%, 80% (weight percentage) $SnCl_2$ in PyDI-5FPE, the calculated volumetric heat capacities are 1.40, 1.42, 1.44, 1.48 and 1.54, as shown in Fig. 4..

Figure S10 shows the measured thermal conductivity as a function of the sample size (R_{pp}) for the samples containing 20-80 wt% $SnCl_2$. As expected, the measured thermal conductivity shows large variation at small sampling size and small variation at large sampling size. Clearly, all the measurement converged at R_{pp} = 70.9 μm. The converged thermal conductivity at the four different sampling locations is then averaged again to obtain the final thermal conductivity data shown in Fig. 4 of the paper.

References

[1] J.M Themlin, M. Chtaib, L. Henrad, P. Lambin, J. Darville, and J.M. Gilles, Characterization of tin oxides by x-ray-photoemission spectroscopy, *Phys. Rev. B* **1992**, 46, 2460-2466.

[2] H. Seema, K. C. Kemp, V. Chandra, and K. S. Kim, Graphene-SnO2 composites for highly efficient photocatalytic degredation of methylene blue under sunlight, *Nanotechnology* **2012**, 23, 355705.

[3] Y. Jung, W. Yang, C. Y. Koo, K. Song, and J. Moon, High performance and high stability low temperature aqueous solution-derived Li-Zr co-doped ZnO thin film transistors, *J. Mater. Chem.* **2012**, 22, 5390-5397.
[4] X. Q. Pan, and L. Fu, Oxidation and phase transitions of epitaxial tin oxide thin films on sapphire, *J. Appl. Phys.* **2001**, 89, 6048-6055.
[5] L. Y. Liang, Z. M. Liu, H. T. Cao, and X. X. Pan, Microstructural, optical, and electrical properties of SnO thin films prepared on quartz via a two-step method, *Appl. Mater. & Interf.* **2010**, 2, 1060-1065.
[6] A. Parveen, K. R. Anilkumar, S. D. Patil, and A. S. Roy, PEO/$SnCl_2$/PANI composites: As an electrolyte in solid-state battery. *Ionics* **2013**, 19, 91-97.
[7] V. S. Marakatti, G. V. Shanbhaq, and A. B. Halgeri, Condensation reactions assisted by acidic hydrogen bonded hydroxyl groups in solid tin(II)hydroxychloride, *RSC Adv.* **2013**, 3, 10795-10800.
[8] K. Chen, and D. Xue, Crystallization of tin chloride as a promising pseudocapacitor electrode, *Cryst. Eng. Comm.* **2014**, 16, 4610-4618.
[9] J. Liu, J. Zhu, A. Schmidt, and R.G. Yang, Simultaneous measurement of thermal conductivity and heat capacity of bulk and thin film materials using frequency-dependent transient thermoreflectance method, *Rev. Sci. Instrum.* **2013**, 84, 034902.
[10] D.G. Cahill, Analysis of heat flow in layered structures for time-domain thermoreflectance, *Rev. Sci. Instrum.* **2004**, 75, 5119.
[11] A.J. Schmidt, X. Chen, and G. Chen, Pulse accumulation, radial heat conduction, and anisotropic thermal conductivity in pump-probe transient thermoreflectance, *Rev. Sci. Instrum.* **2008**, 79, 114902.
[12] J. Zhu, D. Tang, W. Wang, J. Liu, K. W. Holub, and R.G. Yang, Ultrafast thermoreflectance techniques for measuring thermal conductivity and interface thermal conductance of thin films, *J. Appl. Phys.* **2010**, 108, 094315.
[13] Y. Touloukian, and E. Buyco, Thermophysical properties of matter - the TPRC data series.Volume 5. Specific Heat - nonmetallic solids, **1970**.
[14] R. C. Weast, M. J. Astle, and W. H. Beyer, CRC handbook of chemistry and physics, CRC press Boca Raton, FL, **1988**, 69.
[15] M. J. Assael, S. Botsios, K. Gialou, and I. N. Metaxa, Thermal conductivity of polymethyl methacrylate (pmma) and borosilicate crown glass, *Int. J. Thermophys.* **2005**, 26, 1595-1605.

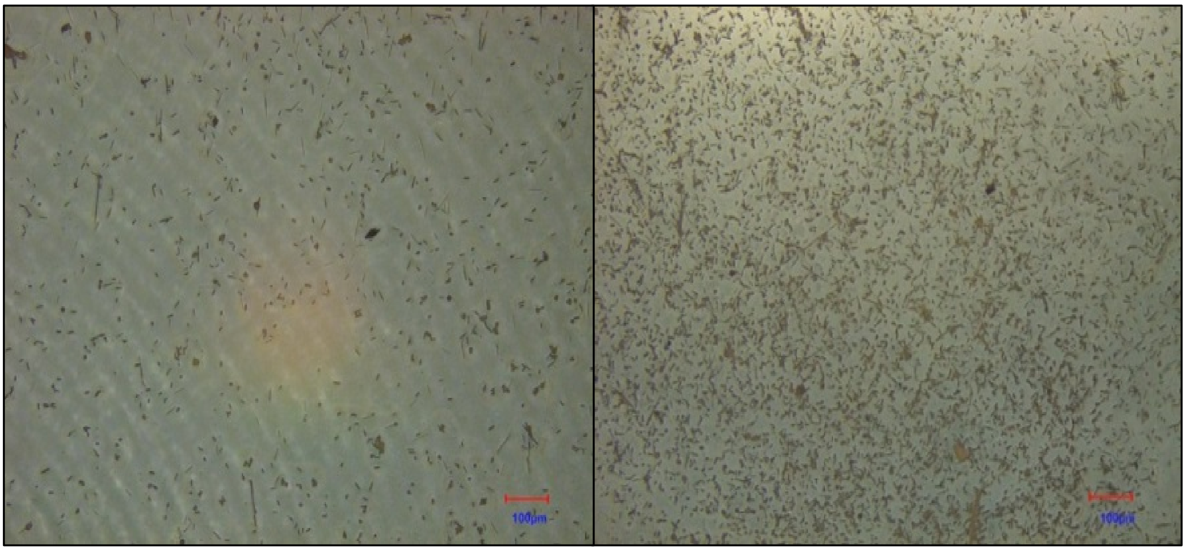

Figure S1. Optical microscope images of 60wt% $SnCl_2$ (left) and 70wt% $SnCl_2$ (right) in PYDI-5FPE polymer, obtained by spin-casting. Red scale bars are 100 microns.

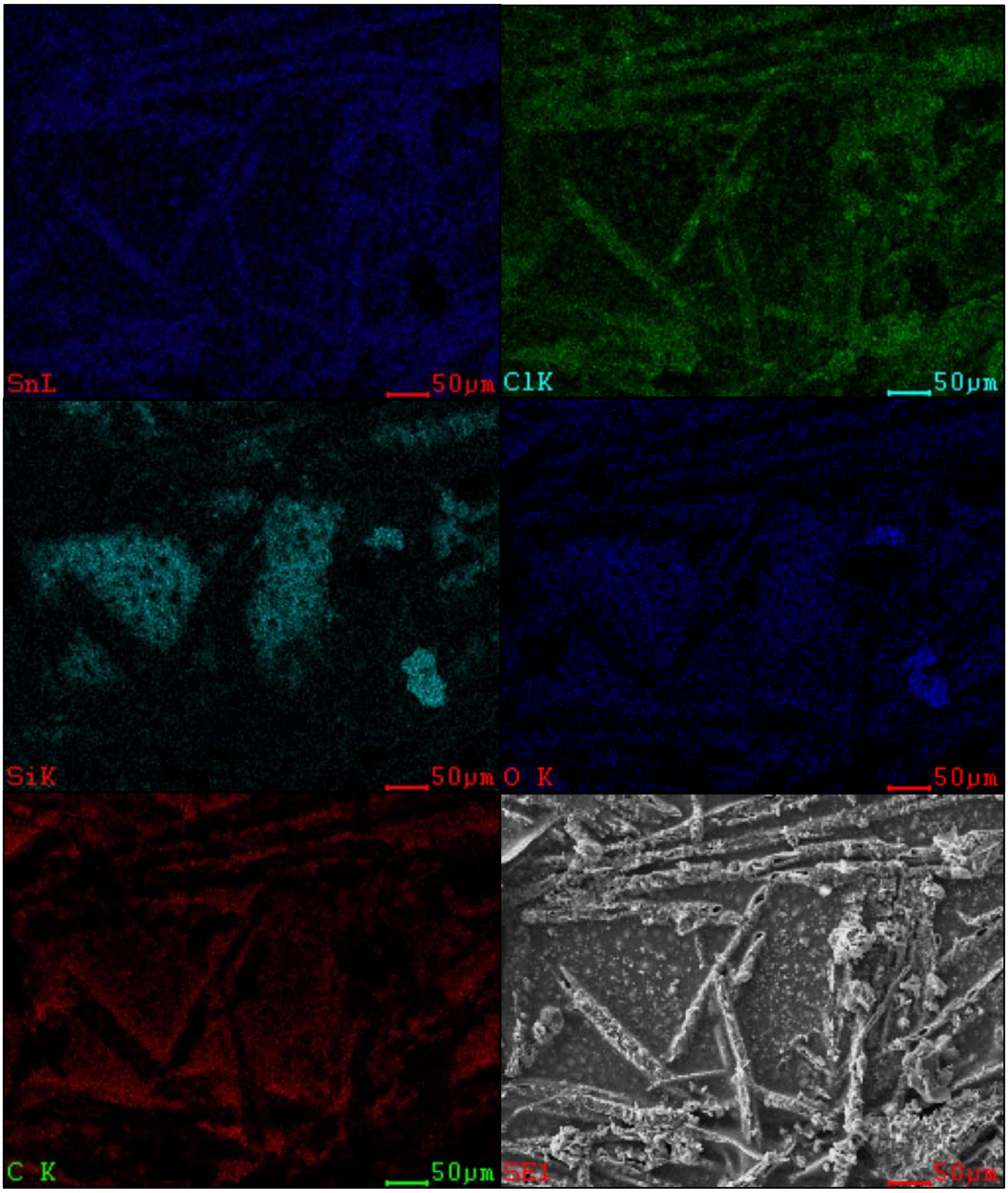

Figure S2. Elemental maps obtained by EDS using an SEM backscatter detector show the coverage of Sn, Cl, Si, O, and C atoms for 60wt% $SnCl_2$ in PYDI-5FPE polymer obtained by drop-casting. Bottom right is the original SEM image.

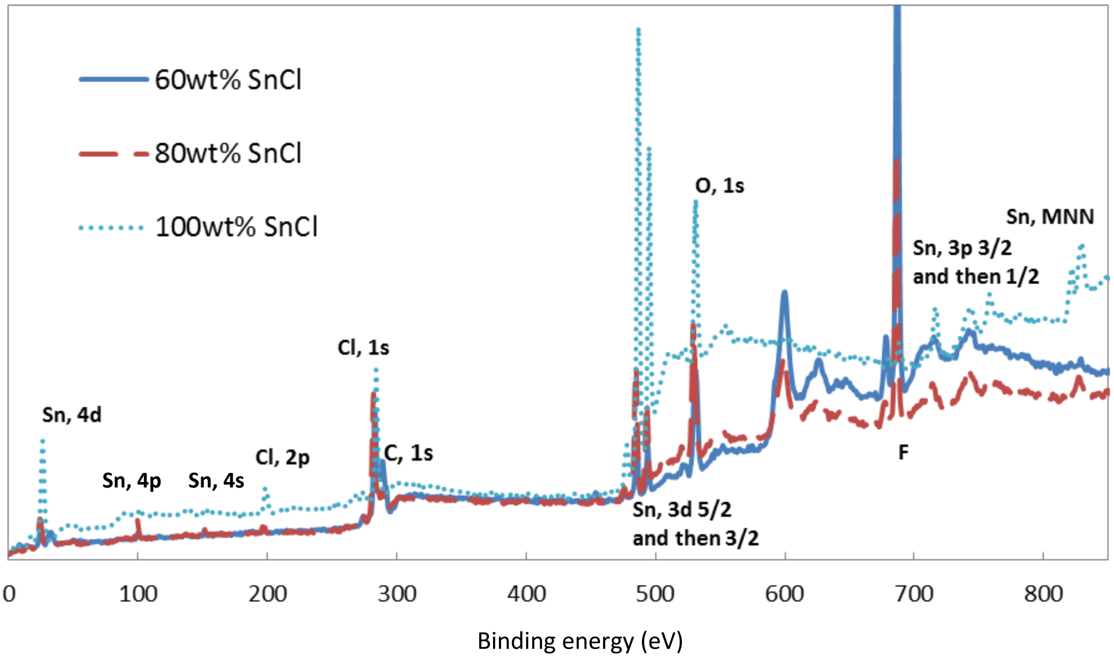

Figure S3. XPS of 60 and 80 wt% tin(II) chloride blended in PYDI-5FPE polymer matrix, and for pure tin(II) chloride film.

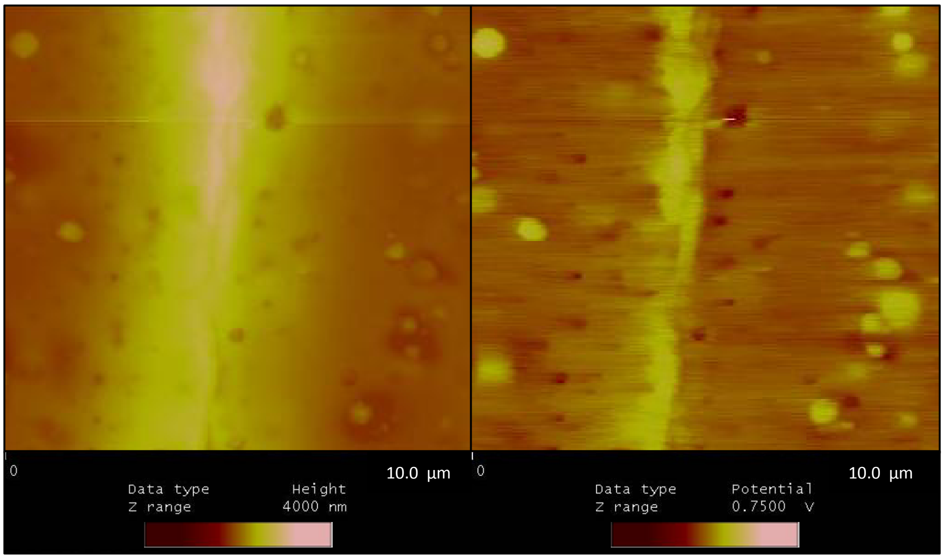

Figure S4. 2D Kelvin probe scans (10 x 10 µm) of height (left) and surface potential (right) for a uniaxial $SnCl_2$ particle (aligned top to bottom) in PYDI-5FPE polymer matrix.

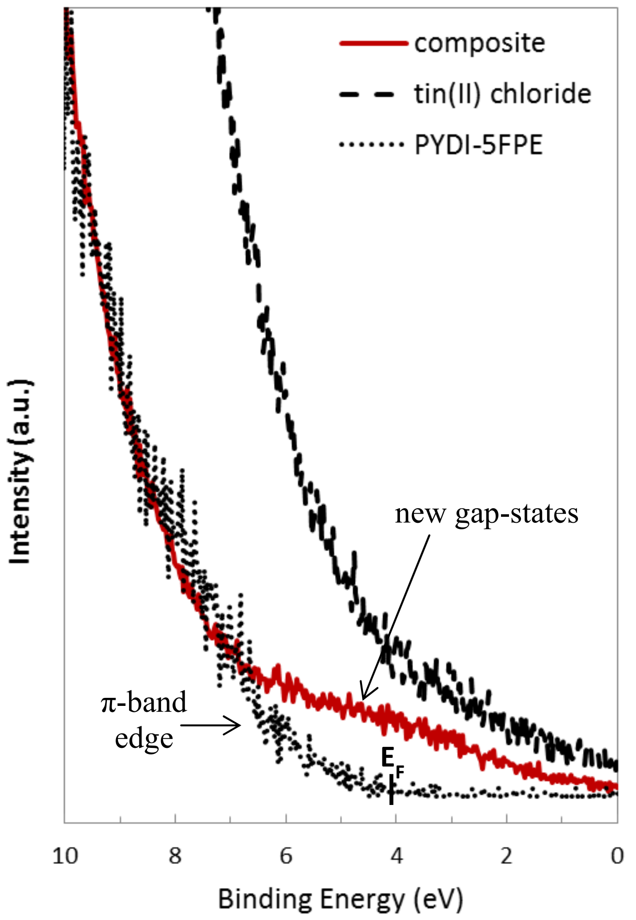

Figure S5. Ultraviolet photoelectron spectra of leading edge of valence band for PYDI-5FPE polymer, tin(II) chloride, and polymer composite with 60wt% $SnCl_2$.

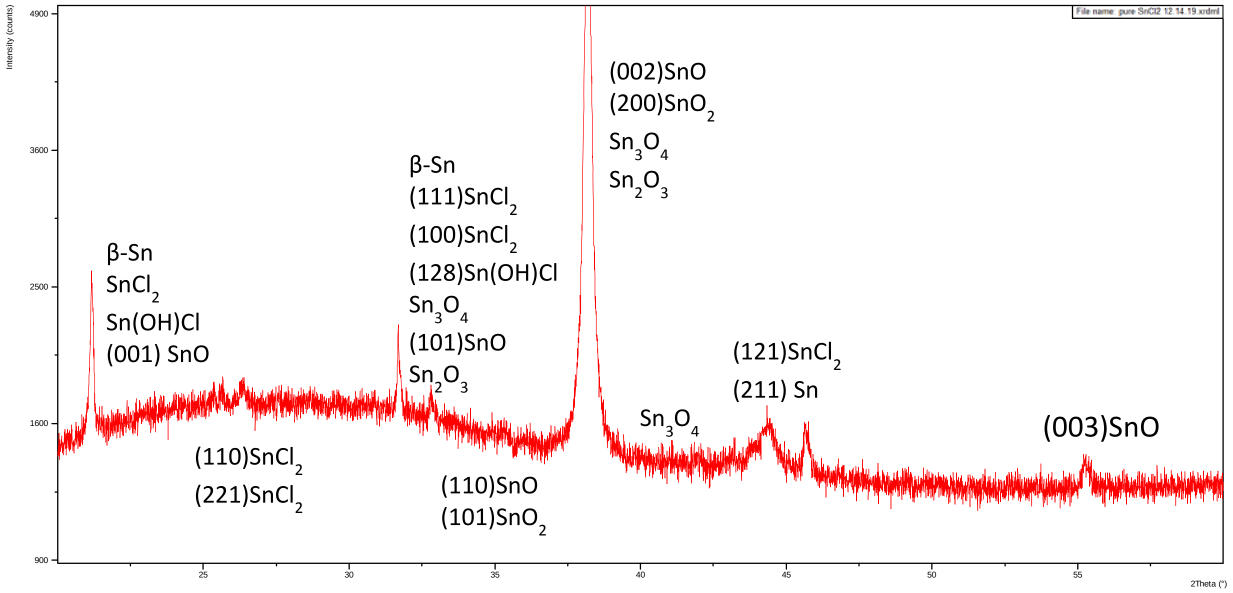

Figure S6. X-ray diffraction shows that pure $SnCl_2$ films are mostly amorphous, with very small peaks for metallic tin, and possibly tin(II) chloride or tin oxide.

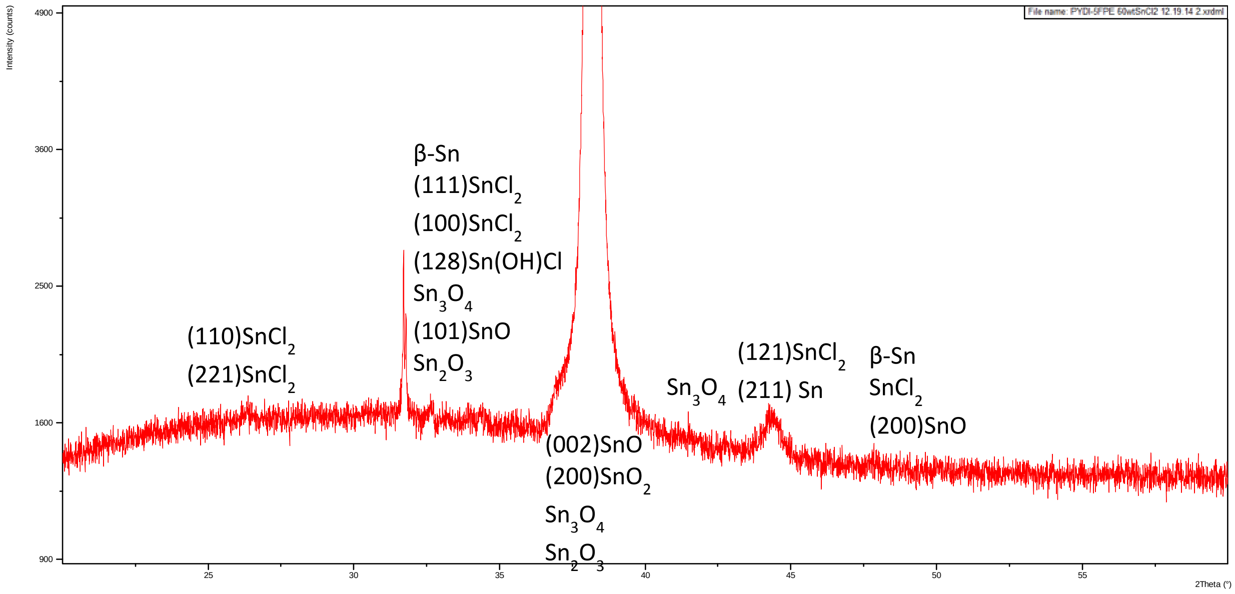

Figure S7. X-ray diffraction shows that composite films are mostly amorphous, with small peaks for metallic tin, tin(II) chloride, and/or tin oxide.

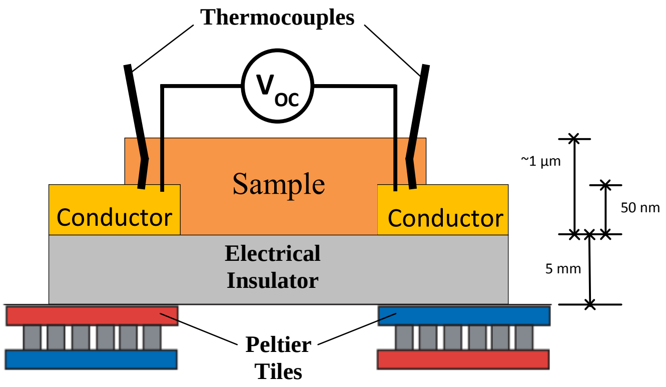

Figure S8. Side-view schematic of S measurement. The length of electrodes is 10 mm (into the page) and width between electrodes is < 2 mm, electrodes are 50 nm thick.

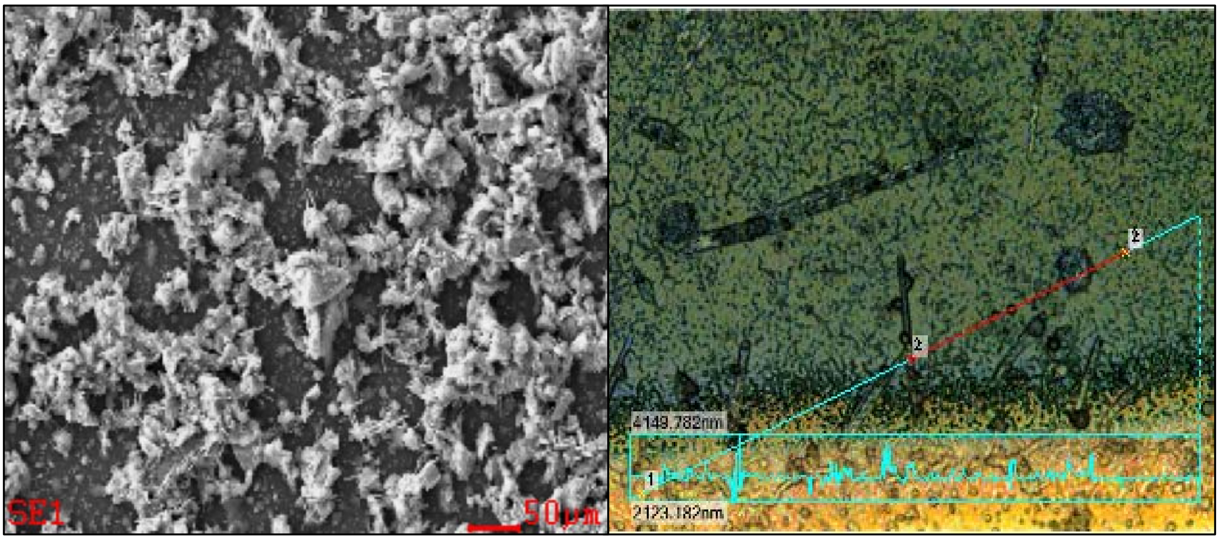

Figure S9. Scanning electron micrograph of 100 wt% $SnCl_2$ drop-casted onto glass with pre-patterned gold electrodes (left), and optical micrograph of anhydrous $SnCl_2$ powder sublimed onto substrates (right).

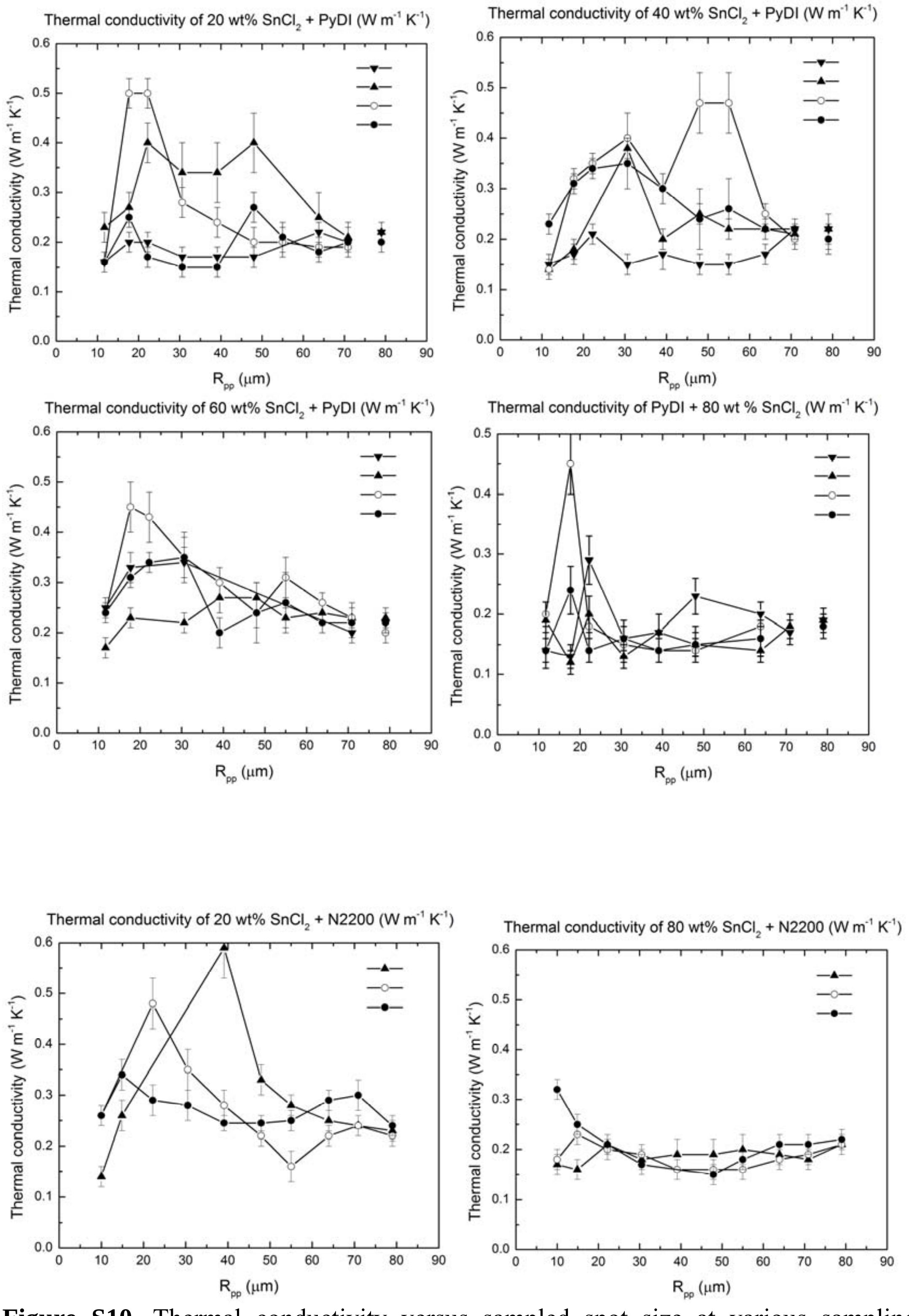

Figure S10. Thermal conductivity versus sampled spot size at various sampling locations (denotes by various symbols in the plots) for various $SnCl_2$ weight percentages.

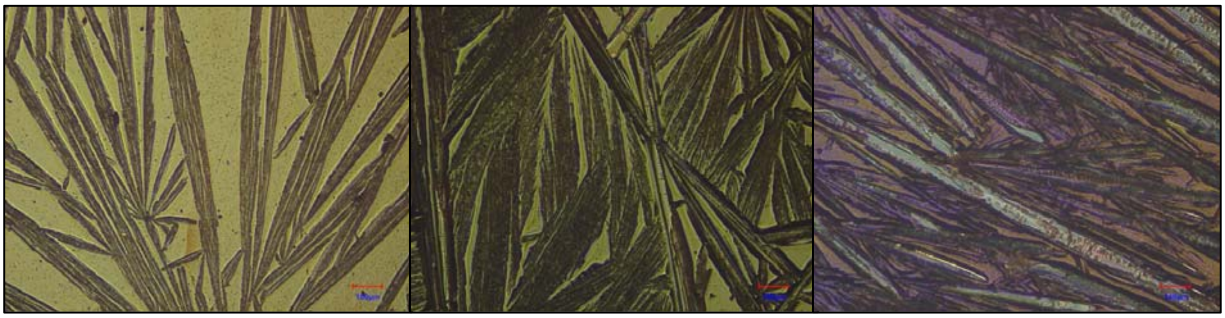

Figure S11. Optical microscope images of PBTTT-C14 blended with 20, 60, and 80 wt% cobalt(III) acetylacetonate (from left to right). Scale bars are all 100 µm.

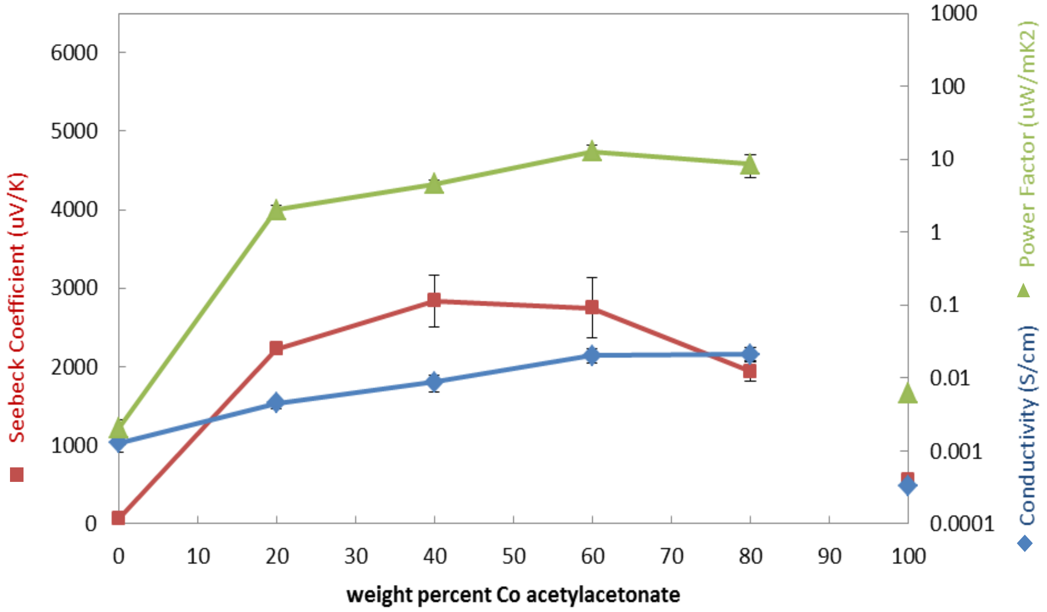

Figure S12. The linear Seebeck coefficient, log scale electrical conductivity, and log scale power factor are plotted versus concentration of starting Co((acac)3) in PQT12 obtained by drop-casting. Values are the average of at least 5 samples.

a)

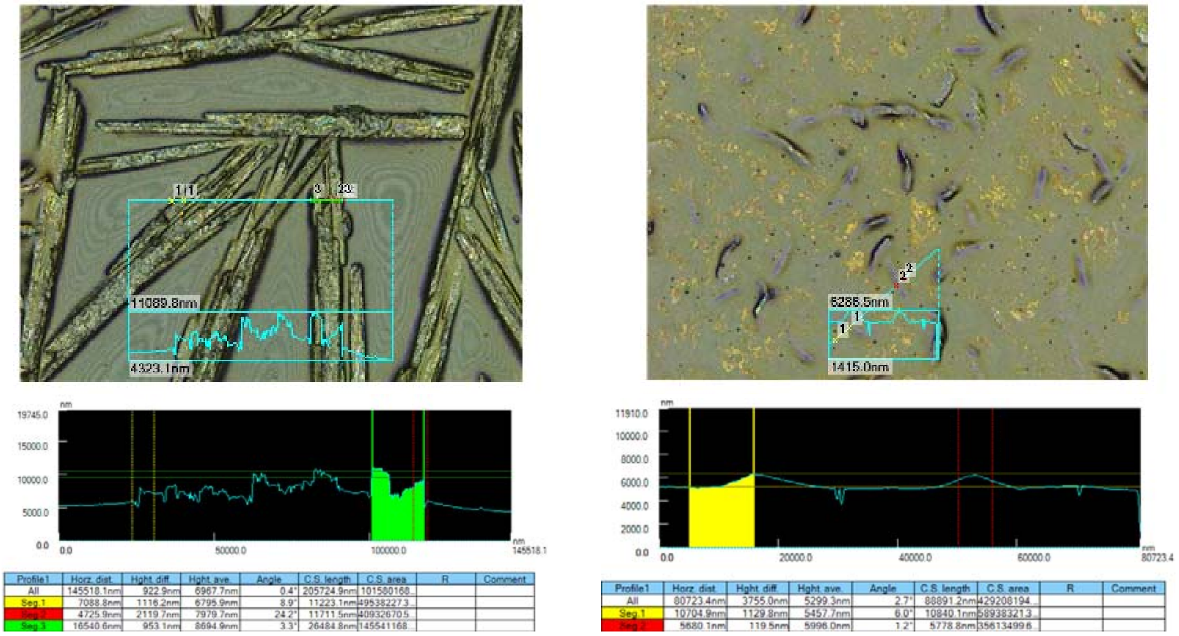

Figure S13. Surface profiles measured by laser optical microscope of 60 wt% $SnCl_2$/PyDI-5FPE polymer composites fabricated by a) drop-casting and b) spin-casting.

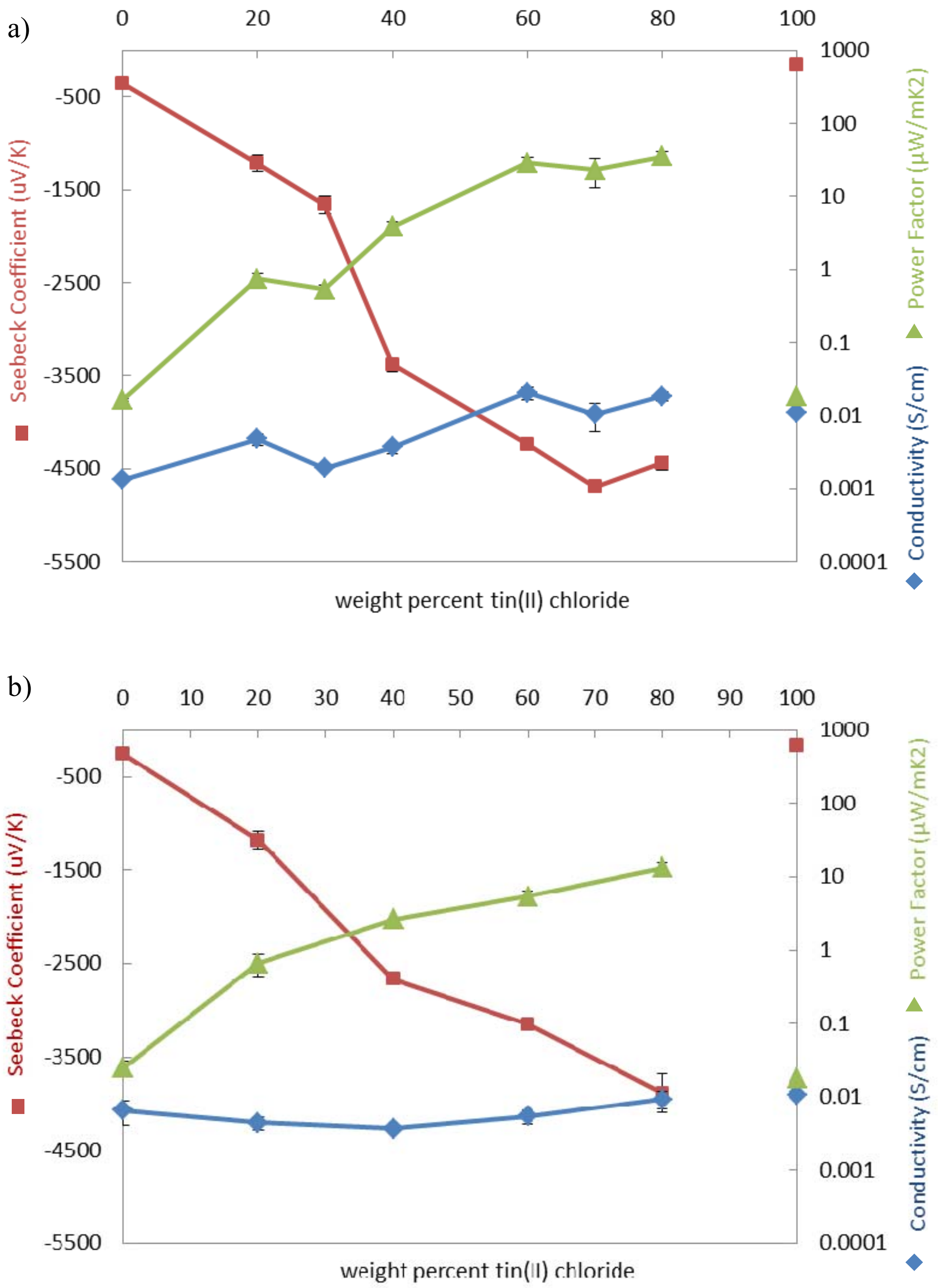

Figure S14. The Seebeck coefficient, electrical conductivity, and power factor are plotted versus concentration of initial tin(II) chloride precursor within polymer matrices and spun-cast for a) PYDI-5FPE and b) P(NDI2OD-T2) composites. Values are the average of at least 5 samples.

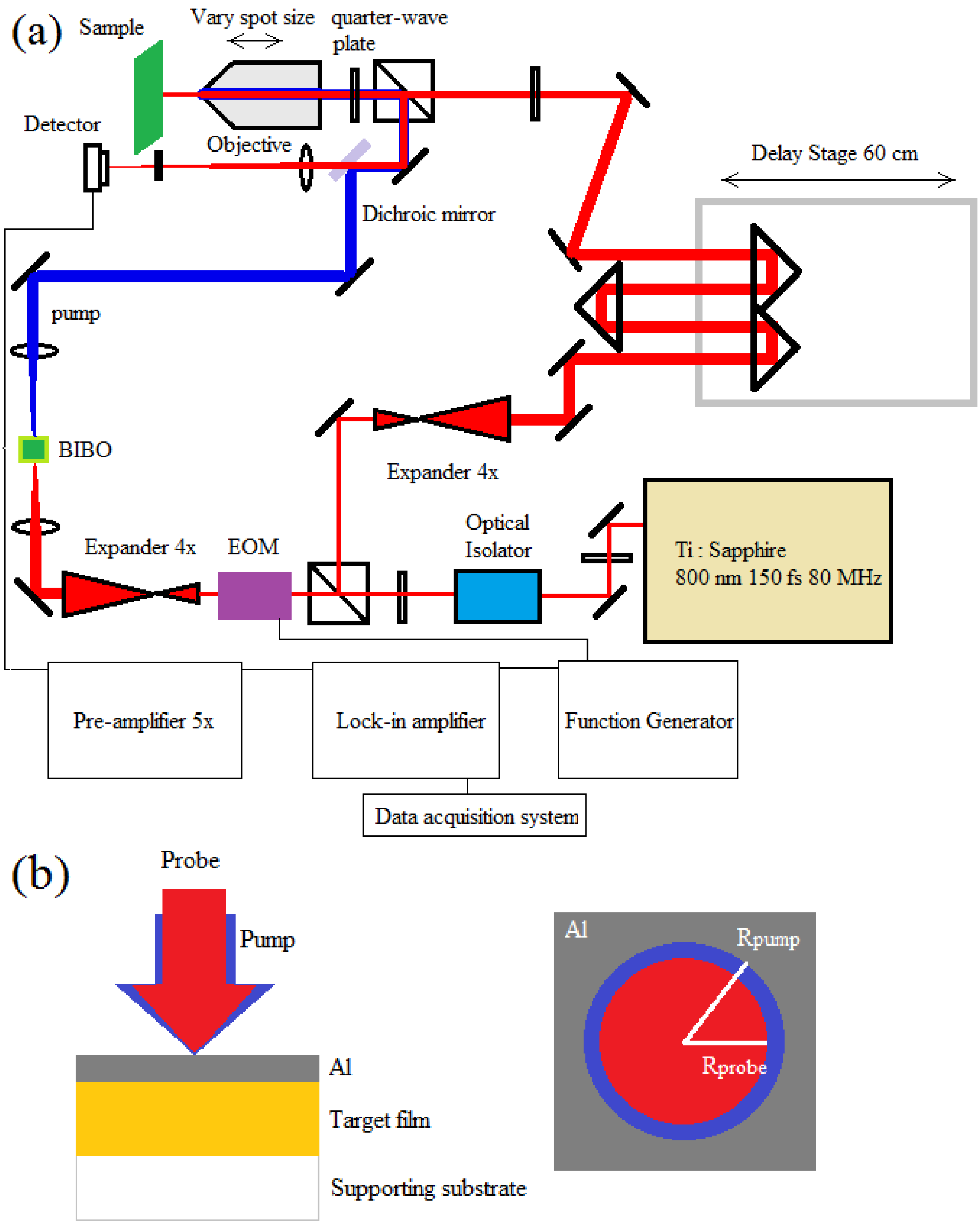

Figure S15. (a) Schematics of the transient thermoreflectance system using femtosecond laser for measurement of thermal conductivity and interface thermal conductance. (b) Schematics of configuration of the sample where Al thin film is deposited on the target film serving as the transducer for temperature measurement.

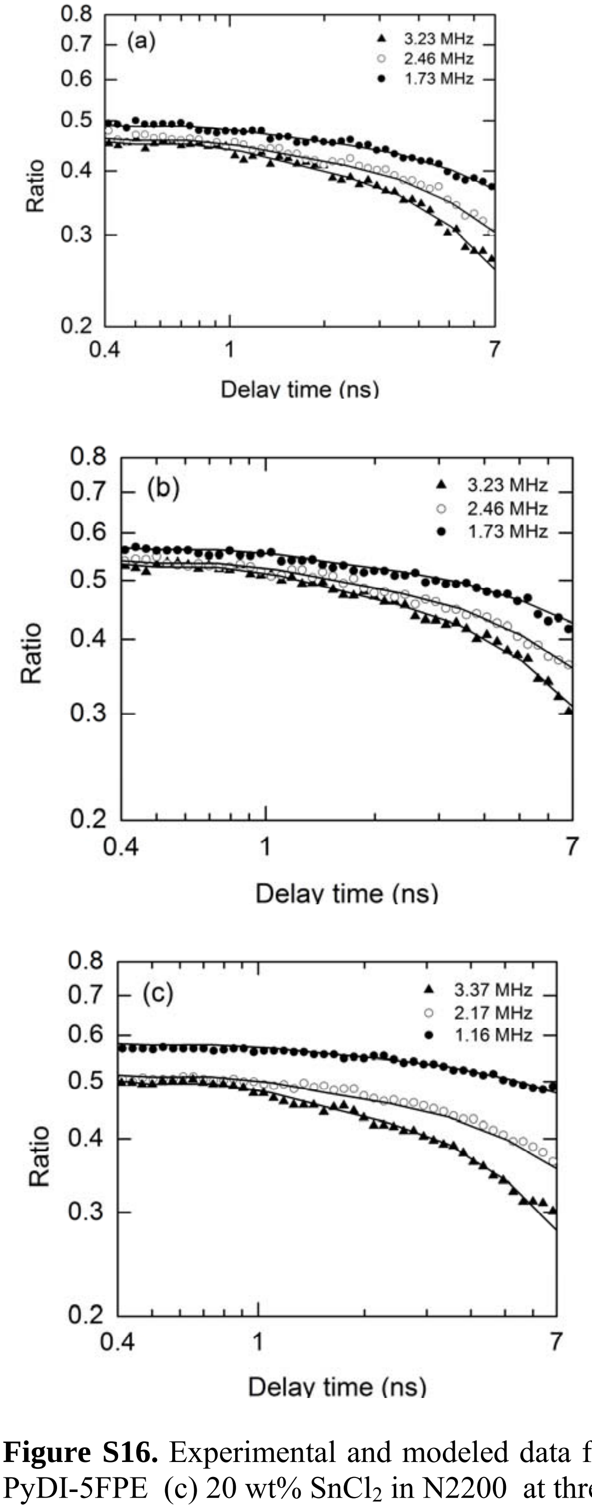

Figure S16. Experimental and modeled data for drop-cast (a) PyDI-5FPE (b) 20 wt% $SnCl_2$ in PyDI-5FPE (c) 20 wt% $SnCl_2$ in N2200 at three modulation frequencies

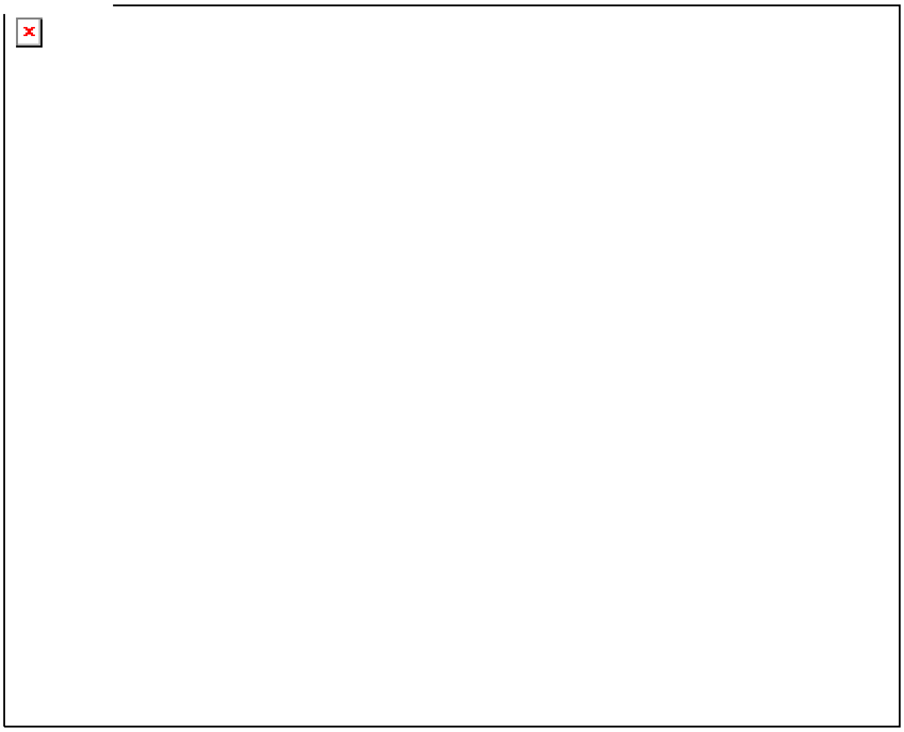

Figure S17. Thermal conductivity versus sampling size (R_{pp}) for SiO_2, PMMA and PyDI-5FPE.

We investigated the suitability of the mixing rule and the Nielsen model of thermal conductivity (Ind. Eng. Chem. Fundam., 13(1), 17-20 (1974)) to our system, as the electronic phenomena may be too localized or dependent on chemical interactions near interfaces for the effective media models to be applicable. Figure S18 shows the predicted thermal conductivity for the P1/$SnCl_2$ composites via the mixing rule and the Nielsen model as well as the experimental data as a function of weight and volume fraction of $SnCl_2$ in P1. We use 0.5 W/mK for the thermal conductivity of pure $SnCl_2$ based on maxima in our spot size-dependent measurements (Figure S10), which is consistent with the expected values of 0.3-0.6 W/mK for metal dichlorides in the literature although no direct measurement of $SnCl_2$ is available yet.

Our experimental data are consistent with both the mixing rule model and the Nielson model for up to 40-60 wt % (20-30 volume %) $SnCl_2$. Above 40 wt % $SnCl_2$, the $SnCl_2$ inclusions develop highly anisotropic morphologies and better-defined interfaces. At this point, the models become unsuitable because of possible increasing interface density and increasing anisotropy in the physical properties, neither of which are accounted for in these models.

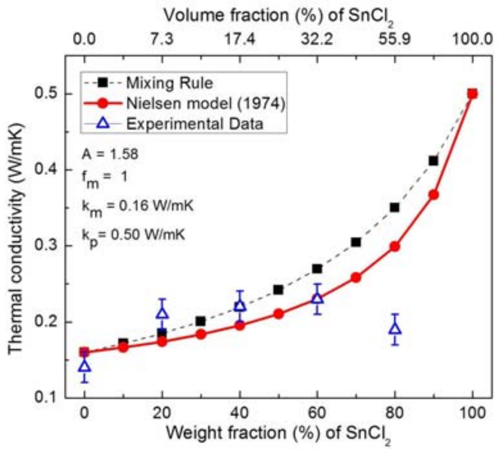

Figure S18. Comparison of the theoretical predictions via mixing rule and Nelson model as well as experimental date for the thermal conductivity of a polymer composite with $SnCl_2$ crystals, as a function of the weight and volume fraction. 100% $SnCl_2$ is assumed to be 0.5 W/mK, based on the highest values we observed for small spot sizes in composite measurements of Figure S10.

Further explanation of the models is as follows. The effective thermal conductivity (k_{eff}) of composites can be obtained:

$$\frac{k_{eff}}{k_m} = \frac{1+ABf}{1-B\psi f} \quad , \tag{1}$$

$$B = \frac{k_p / k_m - 1}{k_p / k_m + A} \quad , \tag{2}$$

$$\psi = 1 + \frac{(1-f_m)}{f_m^2} f \; , \tag{3}$$

where k_m and k_p represent the intrinsic thermal conductivity of the polymer matrix and $SnCl_2$ microstructures, respectively. A and B are variables that primarily depends on the thermal properties and the geometry of matrix and particles. Here, we used 4.93, the value suggested for rods of 10:1 aspect ratio in the above reference. The factor ψ is determined by the volume fraction of dispersed second phase, f, and the maximum packing volume fraction, f_m. The upper bound of Nielsen mode is when A → ∞ and f_m = 1, where the model will become the ordinary mixing rule, i.e., $k_{eff} = k_m \cdot (1-f) + k_p \cdot f$. The value of A and ψ for various types of composites can be obtained in the above reference.
